# Jointly Optimized Deep Neural Networks to Synthesize Monoenergetic Images from Single-Energy CT Angiography for Improving Classification of Pulmonary Embolism

**DOI:** 10.3390/diagnostics12051224

**Published:** 2022-05-13

**Authors:** Matthias A. Fink, Constantin Seibold, Hans-Ulrich Kauczor, Rainer Stiefelhagen, Jens Kleesiek

**Affiliations:** 1 Clinic for Diagnostic and Interventional Radiology, Heidelberg University Hospital, Im Neuenheimer Feld 420, 69120 Heidelberg, Germany; hans-ulrich.kauczor@med.uni-heidelberg.de; 2 Translational Lung Research Center (TLRC), German Center for Lung Research (DZL), Heidelberg University Hospital, 69120 Heidelberg, Germany; 3 Department of Computer Science, Karlsruhe Institute of Technology (KIT), 76131 Karlsruhe, Germany; constantin.seibold@kit.edu (C.S.); rainer.stiefelhagen@kit.edu (R.S.); 4 Institute for AI in Medicine (IKIM), University Hospital Essen, 45131 Essen, Germany; jens.kleesiek@uk-essen.de

**Keywords:** artificial intelligence, deep learning, image-to-image translation, dual-energy computed tomography, pulmonary embolism, emergency radiology

## Abstract

Detector-based spectral CT offers the possibility of obtaining spectral information from which discrete acquisitions at different energy levels can be derived, yielding so-called virtual monoenergetic images (VMI). In this study, we aimed to develop a jointly optimized deep-learning framework based on dual-energy CT pulmonary angiography (DE-CTPA) data to generate synthetic monoenergetic images (SMI) for improving automatic pulmonary embolism (PE) detection in single-energy CTPA scans. For this purpose, we used two datasets: our institutional DE-CTPA dataset $D_{1}$, comprising polyenergetic arterial series and the corresponding VMI at low-energy levels (40 keV) with 7892 image pairs, and a 10% subset of the 2020 RSNA Pulmonary Embolism CT Dataset $D_{2}$, which consisted of 161,253 polyenergetic images with dichotomous slice-wise annotations (PE/no PE). We trained a fully convolutional encoder-decoder on $D_{1}$ to generate SMI from single-energy CTPA scans of $D_{2}$, which were then fed into a ResNet50 network for training of the downstream PE classification task. The quantitative results on the reconstruction ability of our framework revealed high-quality visual SMI predictions with reconstruction results of 0.984 ± 0.002 (structural similarity) and 41.706 ± 0.547 dB (peak signal-to-noise ratio). PE classification resulted in an AUC of 0.84 for our model, which achieved improved performance compared to other naïve approaches with AUCs up to 0.81. Our study stresses the role of using joint optimization strategies for deep-learning algorithms to improve automatic PE detection. The proposed pipeline may prove to be beneficial for computer-aided detection systems and could help rescue CTPA studies with suboptimal opacification of the pulmonary arteries from single-energy CT scanners.

## 1. Introduction

Pulmonary embolism (PE) is a potentially life-threatening condition and represents the third most frequent cardiovascular disease after acute coronary syndrome and stroke [1,2]. Early and accurate diagnosis of PE helps in appropriate risk stratification and could substantially improve treatment outcomes [3]. Because of fast image acquisition protocols and high sensitivity in clot detection, computed tomography pulmonary angiography (CTPA) has become the first-line imaging modality in the diagnostic workup for patients with suspected PE [4,5,6]. However, individual patient-related parameters, such as cardiac function, circulation time, and an increased pulmonary inflow of unopacified blood, known as transient interruption of contrast, can compromise image quality of the CTPA study, sometimes rendering the examination useless for an adequate diagnostic evaluation [7,8]. Detector-based spectral dual-energy CT (DECT) has gained increasing importance in clinical routine because of various post-processing algorithms which allow the reconstruction of energy- and material-selective images from spectral data. DECT enables the creation of discrete acquisitions at different energy levels, resulting in virtual monoenergetic images (VMI) that can mimic low (at high keV) to high (at low keV) iodine-based contrast-enhanced studies. It was shown that an improved iodine attenuation by VMI at lower keV levels enables better delineation and diagnostic accuracy in PE detection and may help rescue CTPA studies with suboptimal opacification of the pulmonary arteries [6]. Since most existing CT datasets consist of conventional single-energy CT scanners, they do not provide spectral information to calculate VMI. Recent studies have proposed deep-learning models to produce high-quality approximations of DECT-derived VMI to overcome these issues [9,10,11]. However, while existing image translation methods can generate visually appealing results, they do not necessarily enforce features that enable the correct identification of certain classes.

In this study, we aimed to develop a jointly optimized end-to-end learnable framework that combines the training of two convolutional neural networks for image translation and downstream PE classification. For this task, we investigated several state-of-the-art image translation methods to predict synthetic monoenergetic images (SMI) for subsequent training of the classification network. We evaluated the proposed pipeline on an independent external test set comprising single-energy CT data with slice-wise annotations for PE presence by domain medical experts and compared it against other naïve classification approaches.

## 2. Materials and Methods

### 2.1. Datasets and Study Design

**Figure 1:**
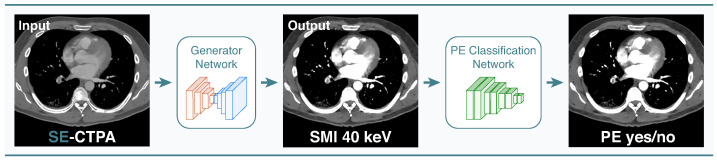
Proposed joint optimization framework. Single-energy CT pulmonary angiography (SE-CTPA) arterial series are translated into synthetic monoenergetic images (SMI) using a L1 loss-based ResNet encoder-decoder convolutional network. The SMI are then processed for pulmonary embolism (PE) classification using a ResNet50 convolutional neural network.

Two datasets were used for this study. Institutional anonymized DE-CTPA data $D_{1}$ were retrospectively included from 27 consecutive adult patients suspected of having PE, referred from 15 July to 15 August 2020, during routine clinical workup in our radiology department at Heidelberg University Hospital. No exclusion criteria were defined. Institutional DE-CTPA scans were performed on a dual-layer detector CT (IQon Spectral CT, Philips Healthcare, Hamburg, Germany), from which standard arterial series and the corresponding VMI at low-energy levels (40 keV) were reconstructed, yielding a final dataset of 7892 image pairs without information on PE occurrence. The second dataset $D_{2}$ was a subset of the 2020 RSNA Pulmonary Embolism CT Dataset, the largest publicly available, expert-annotated dataset of CTPA studies to date [12]. Of the 7279 annotated CTPAs from $D_{2}$, we sampled 10% of the training data. The final dataset $D_{2}$ consisted of a total of 161,253 PE-annotated slices with roughly the same label distribution as present in the open training set. A general overview of the proposed image translation and classification framework is shown in Figure 1.

**Figure 2:**
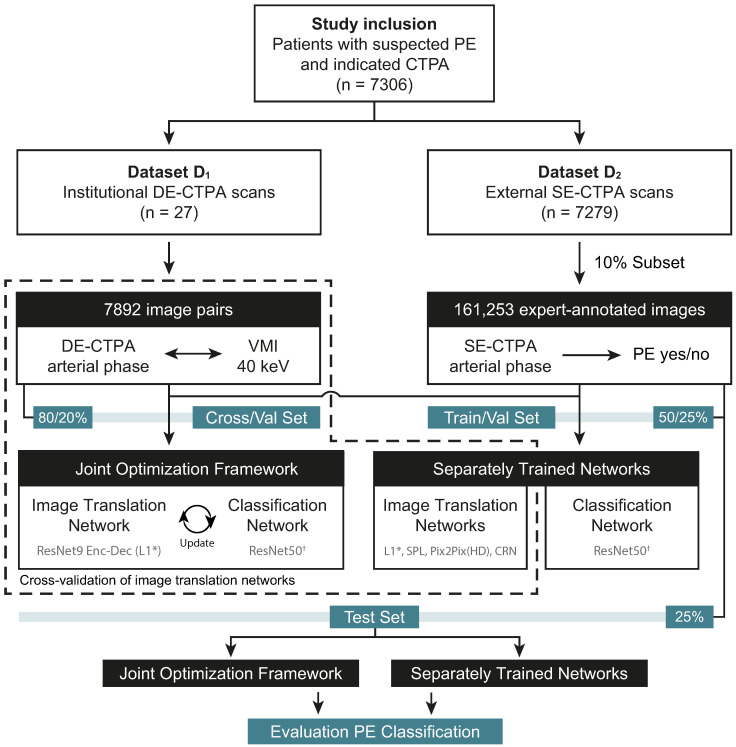
Flowchart of study sample. ${}^{*}$L1 loss-based image translation and ${}^{†}$ResNet50 classification networks with identical architecture. CTPA = CT pulmonary angiography, DE = dual-energy, PE = pulmonary embolism, SE = single-energy, VMI = virtual monoenergetic images.

For our experiments, we considered the scenario where we are given these two distinct datasets $D_{1}$ and $D_{2}$. $D_{1}$ comprised unannotated images with DECT polyenergetic and corresponding 40 keV monoenergetic depictions but no information on the occurrence of PE. $D_{2}$ contained a set of conventional single-energy CTPA (SE-CTPA) images with slice-wise binary PE annotations (PE/no PE) without corresponding monoenergetic representations. To take advantage of the DECT technology, we aimed to design a unified model that would jointly optimize disease identification and domain adaption most fitting for the task. We have formulated these two tasks in the same framework, so that (a) it trains these tasks end-to-end, and (b) the two tasks can be mutually beneficial. We compared our optimized framework against the same two but separately trained networks and further straightforward approaches using other state-of-the-art image translation methods, all of which were finally evaluated in the same downstream PE classification task. Figure 2 summarizes the study design.

### 2.2. Model Development

**Figure 3:**
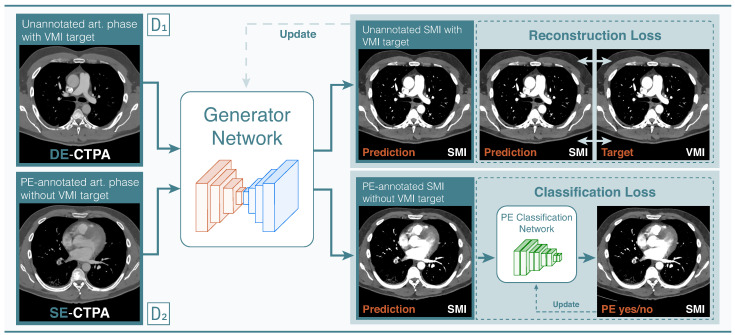
End-to-end learnable image translation and classification pipeline. The ResNet9 encoder-decoder network (Generator Network) was trained on $D_{1}$ to predict synthetic monoenergetic images (SMI). Using the trained generator network, the annotated SE-CTPA images from $D_{2}$ were translated into SMI, which were then fed into a ResNet50 convolutional network (PE Classification Network) for training PE classification. The generator and classification networks were updated by a reconstruction and classification loss, respectively.

We developed our jointly optimized framework using a two-stage approach: first, an image translation model (Generator Network) was trained using the images of $D_{1}$, afterwards a classification network (PE Classification Network) was trained on $D_{2}$ using the image translation model’s outputs as the classification networks inputs. We trained our networks jointly in an end-to-end manner by sequentially passing data through the Generator and PE Classification Network (Figure 3).

#### 2.2.1. The Generator and PE Classification Network

Our Generator Network follows the official Pytorch implementation of the fully convolutional 9-block ResNet encoder-decoder network [13,14]. As such, three strided convolutions with BatchNorm [15] and ReLU activations encode the image input and feed these to nine residual blocks [16]. Three transposed convolutions [17] are used to produce the output image. For our PE Classification Network, we used the common ResNet50 architecture [16].

#### 2.2.2. Joint Optimization of the Generator and Classification Network

The proposed framework jointly optimizes two tasks in an end-to-end manner [11]. As one task, we considered the problem of translating between the domain of polyenergetic x∈X and VMI images y∈Y as a paired image translation problem. Here, the generator aims to learn a mapping *G*:x→y, which minimizes the difference between the two paired images. The generator consists of a fully convolutional neural network which allows a pixel-wise regression towards VMI images. This objective can be expressed as

(1) $$L_{L1}=E_{x,y}[||G\left(x\right)-{y||}_{1}].$$

We used the mean absolute error as it has been found to lead to less blurry images [13]. Consecutively, the output of the generator was fed into a classification network *C*, which attempts to predict the occurrence of a disease label *z*, *C*: G(x)→z of the annotated dataset. The classification network consists of a deep convolutional network that takes an image as input and outputs a scalar value. We used a sigmoid activation σ for making output predictions while we dealt with the binary classification task (PE/no PE).

(2) $$\begin{array}{cc} L_{cls}= & E_{x,z}[-z\log\sigma \left(C\left(G\left(x\right)\right)\right)\\ & -(1-z)\log(1-\sigma (C(G(x)))] \end{array}$$

To optimize both objectives during the training process, we constructed our dataset as a combination of the two datasets (Figure 2). During the networks’ optimization process, we sampled the batch in such a way that, on average, it consisted of 50% of either dataset. Therefore, target disease labels appeared for half of the batch and monoenergetic target images for the other half. To accommodate this circumstance into the optimization function, we introduced a marker variable *m* that switches between [0,1] depending on whether we were presented a target image *y* or a target label *z*. In this manner, the final loss can be formulated as

(3) $$L=m\cdot L_{cls}+\left(1-m\right)\cdot L_{L1}.$$

This batch constellation led to a balanced optimization scheme allowing neither objective to dominate the training. For back-propagation of the resulting gradients, we kept one of the networks frozen while updating the other depending on the respective objective. This process behaved similarly to adversarial training. During inference, an SE-CTPA is passed to the generator network, thus, producing an SMI. Using the SMI as input, the classification network then predicts the likelihood of a PE within a range of 0–1.

#### 2.2.3. Training of the Jointly Optimized Framework

We used Adam [18] for optimization with a learning rate of 0.0002, $\beta_{1}=0.9$ and $\beta_{2}=0.99$ with a weight decay of 0.00001. After training for five epochs on the joint dataset, we decayed our learning rate to 0 over the following five epochs. For our purposes, we split each scan slice-wise and used the individual slices for further processing. Each slice image was used as one channel image normalized using the dataset’s mean and standard deviation. We used an image size of 512×512 with a batch size of five for all our experiments. We split the RSNA subset patient-wise 50%/25%/25% into train-, val- and test-sets, respectively. For the cross-validation on our institutional dataset, we used a distinct patient-wise 80%/20% train- and val-set for each split.

#### 2.2.4. Comparison with Other Separately Trained Networks

We compared our end-to-end framework against various image translation algorithms using the same network architecture unless further specified. The L1 loss-based generator describes disjoined training of our generator and classification model. Pix2Pix refers to Isola et al.’s conditional generative adversarial network (GAN), which is optimized using an adversarial and L1 loss [13]. Cascaded refinement networks (CRN) make use of feature matching losses using an external pre-trained network [19]. For the feature matching loss, we applied a visual geometry group (VGG)-network and used the architecture as described by Chen and Koltun [19]. Wang et al.’s Pix2PixHD consists of a multi-scale encoder-decoder architecture optimized using multi-scale adversarial and feature matching losses [20]. The spatial profile loss (SPL) describes an alternative to the L1 loss as its formulation incorporates the images profile structure [21]. We further added L1 losses to feature loss-based methods (CRN, Pix2PixHD), denoted by (*), since we have found the training of these methods to be unstable otherwise.

### 2.3. Statistical Analysis

For model development and training, we used the Pytorch framework (Python, Python Software Foundation) and an NVIDIA GeForce2080 [22]. Statistical analyses were performed using the scikit-learn metrics API version 0.24.2 (Python, Python Software Foundation). To evaluate PE classification performance by the PE Classification Network for different image translation methods, we trained all methods on the same split in the cross-validation setting of our internal dataset. For evaluation of the VMI reconstruction results of the Generator Network, we performed a five-fold cross-validation and averaged our reconstruction results in terms of peak signal-to-noise ratio (PSNR) and a structural similarity index measure (SSIM) [23]. Both metrics compare the projected image to its ground truth target. PSNR is defined via the mean squared error between the two images while SSIM highlights their differences in luminance, contrast, and structure.

Evaluation of the classification performance of the PE Classification Network was performed by a binary classification on slice level for each presented image domain, yielding the area under the curve (AUC) on the test split of the model which performed best on the validation set. Since our classification model output continuous values, with higher values corresponding to higher model reliability regarding the occurrence of PE, we chose a threshold-independent setting to calculate the AUC by weighting the true-positive rate against the false-positive rate across all possible thresholds to indicate the probability by which our classifier preferred a randomly selected PE instance over a negative one. We validated our model after each epoch.

## 3. Results

**Figure 4:**
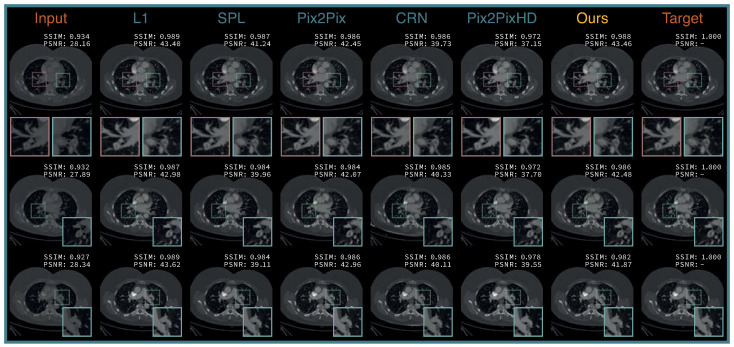
Qualitative comparison of the different image translation methods on our institutional DE-CTPA dataset. The respective structural similarity index measure (SSIM) and peak signal-to-noise ratio (PSNR) values are given in each image. Ours denotes the proposed joint optimization framework.

**Figure 5:**
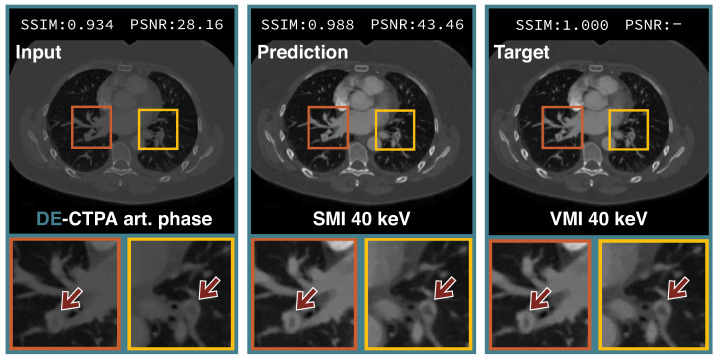
Qualitative samples of our ResNet9 encoder-decoder image translation network. Areas around pulmonary emboli are highlighted and zoomed in the row below. Arrows indicate clot locations in the pulmonary arteries.

The qualitative results on the reconstruction ability of our proposed method and the compared baselines are shown in Figure 4. All tested methods managed to translate the polyenergetic DE-CTPA images into SMI with a higher iodine opacification of the pulmonary arteries, yielding a similar visual appearance compared to the VMI target domain. The DE-CTPA arterial phase, the predicted SMI of our proposed framework, and the VMI target are outlined in Figure 5. Both SMI predictions and VMI reconstructions present higher attenuated pulmonary arteries compared to the polyenergetic arterial phase, with a better delineation of clots in the segmental arteries of both lower lobe arteries (Figure 5, arrows).

Table 1 shows the quantitative SSIM and PSNR values for the original SE-CTPA domain without SMI preprocessing and for the SMI predictions by the separately trained networks and our jointly optimized framework, each with the corresponding PE classification performance of the downstream ResNet50 network.

With the exception of the feature loss-based CRN model, all methods succeeded in producing high-quality SMI predictions. Our method achieved SMI predictions with an SSIM of 0.984 ± 0.002 and a PSNR of 41.706 ± 0.547 dB, revealing a better quantitative image quality than the original arterial SE-CTPA phase and similar visual predictions to the best-performing L1-based generator. Our framework optimized on the image-based comparison and outperformed the feature loss and adversarial methods for the evaluated PE classification task. Despite similar SSIM and PSNR results, the L1 loss-based model generated images that slightly compromised the PE classification performance of the ResNet50 network, while the other compared models degraded the performance. Our proposed method generated visually fitting SMI and achieved improved classification results with an AUC of 0.84 compared to the SE-CTPA baseline and other classification approaches with AUCs up to 0.81.

## 4. Discussion

In this study, we have assessed several state-of-the-art image translation methods for generating synthetic monoenergetic images from single-energy CT scans. We found that, while these dual-energy mapping networks create visually similar predictions to the monoenergetic reconstruction targets, PE classification on these SMI predictions was inferior to that on the original SE-CTPA scans. We extended these methods using a multitask optimization approach, wherein both combined networks achieved better image reconstruction and classification results. External validation of our proposed framework on expert-curated single-energy CTPA scans resulted in an increase in AUC for PE classification from 0.81 to 0.84 compared with other straight forward classification approaches.

We consider this setting relevant since DECT imaging is still not readily available in clinical practice due to complex practical implementations, proprietary patents held by major CT vendors, and the high acquisition costs of the DECT technique compared to conventional SECT scanners, especially in remote healthcare facilities. As part of our clinical routine protocol, we use VMI at the lowest spectrum of monoenergetic reconstructions of 40 keV, as it has been shown to achieve best results in terms of contrast-to-noise and signal-to-noise ratios [24]. VMI reconstructions at 40 keV have been found to improve the quantitative image quality of DE-CTPA studies with suboptimal contrast attenuation of the pulmonary arteries, leading to an increased diagnostic accuracy and confidence in PE detection by radiologists [24,25]. The beneficial effect of using low-keV VMI reconstructions also applies to computer-aided detection (CAD) systems. Recent work has shown that the diagnostic accuracy of a commercially available CAD application had a better performance in PE detection on VMI than on the corresponding dual-energy polyenergetic images, resulting in a significantly lower rate of false-positive PE findings, which argues for the implementation of VMI as the basis for CAD analysis in clinical practice [26,27]. Moreover, previous studies have demonstrated that radiologists’ diagnostic accuracy in detecting PE on CTPA can be improved by CAD systems, although a relatively large number of false-positive results are generated on conventional polyenergetic images [28]. This circumstance still limits the use of automatic detection models in clinical practice and may also be improved by using synthetic monoenergetic data.

To the best of our knowledge, there are no studies evaluating single-energy CT-derived SMI on the performance of CAD systems or its impact on the diagnostic accuracy and confidence of radiologists, especially in SE-CTPA studies with suboptimal contrast attenuation of the pulmonary arteries. This would have practical implications at institutions without DECT scanners, as mapping SE-CTPA series to VMI may allow using these capabilities of DECT technology to rescue diagnostically insufficient, or even non-diagnostic, PE examinations. However, the implementation of CAD algorithms, and the impact of the proposed framework on diagnostic readings by domain medical experts, deserve further exploration in future studies and are beyond the scope of this study.

Our study had limitations. For training the image translation networks, we used only a small number of CTPA studies, each acquired on one type of dual-energy CT scanner with standardized scanning parameters and a defined iodine administration protocol. Although the reconstruction results were of high quality, this approach has potential implications for PE classification performance on external datasets; the use of an inhomogeneous training set from different dual-energy CT scanners and keV levels could lead to further improvements in PE classification. However, we assume good generalizability of the trained model because the CTPA studies in our test set were collected from institutions in five different countries, providing diversity in patient populations, imaging devices, and protocols [12]. Furthermore, we implemented a ResNet50 network for automated PE classification instead of anatomical PE detection on SMI as proof-of-principle to improve the diagnostic performance of our joint optimization approach. Due to the slice-wise binary annotations on PE presence and the absence of bounding boxes, regions of interest, or centroid markers of intraluminal clots in our test set, we were unable to test our model for PE detection performance.

## 5. Conclusions

Our proposed joint optimization strategy allows training of translating polyenergetic into monoenergetic images without losing features necessary for automatic PE classification. Our model represents a noticeable improvement over straightforward classification, while outperforming existing methods. This may prove beneficial in performing high-quality DECT imaging without the conventional hardware-based DECT solutions and may also help to rescue single-energy CTPA studies with low contrast attenuation of the pulmonary arteries for patients with pulmonary embolism.

## Figures and Tables

**Table 1:** Quantitative results and pulmonary embolism classification performance of the jointly optimized framework and separately trained image translation and classification networks.

Domain	SSIM	PSNR	AUC
SE-CTPA	0.945 ± 0.007	30.189 ± 0.690	0.8142
L1	**0.984 ± 0.002**	**42.365 ± 0.642**	0.8102
SPL	0.983 ± 0.002	40.888 ± 0.216	0.8061
Pix2Pix	0.978 ± 0.003	40.897 ± 0.697	0.8051
Pix2PixHD	0.971 ± 0.004	38.739 ± 0.624	–
CRN	0.371 ± 0.551	19.482 ± 16.033	–
Pix2PixHD *	0.971 ± 0.004	38.415 ± 1.278	0.8019
CRN *	0.976 ± 0.005	37.582 ± 1.574	0.8038
Joint Optimization Framework	**0.984 ± 0.002**	41.706 ± 0.547	**0.8420**

Data are mean ± standard deviation. Best results in bold. * Added L1 losses to feature loss-based methods. AUC = area under the receiver operating characteristic curve, SE-CTPA = single-energy CT pulmonary angiography, SSIM = structural similarity index measure, PSNR = peak signal-to-noise ratio.

## Data Availability

Restrictions apply to the availability of the DECT data used to train the image-to-image translation models because of institutional policies. The RSNA Pulmonary Embolism CT Dataset is composed of CT pulmonary angiograms and annotations related to pulmonary embolism. It is available at https://www.rsna.org/education/ai-resources-and-training/ai-image-challenge/rsna-pe-detection-challenge-2020 (accessed on 15 April 2022).

## Abbreviations

The following abbreviations are used in this manuscript:

AUC	Area under receiver operating characteristic curve
CRN	Cascaded refinement network
CTPA	CT pulmonary angiography
DECT	Dual-energy CT
DE-CTPA	Dual-energy CT pulmonary angiography
keV	Kiloelectronvolt
PE	Pulmonary embolism
PSNR	Peak signal-to-noise ratio
SE-CTPA	Single-energy CT pulmonary angiography
SMI	Synthetic monoenergetic image
SPL	Spatial profile loss
SSIM	Structural similarity index measure
VGG	Visual geometry group
VMI	Virtual monoenergetic image
